# Comparative antisickling and antioxidant activities of *Pseudobombax ellipticum* cultivars in relation to their metabolite profiling using LC/MS

**DOI:** 10.1039/d3ra03312k

**Published:** 2023-07-14

**Authors:** Ahmed S. Mohamed, Omnia Y. Abd El Dayem, Ali M. El Shamy, Fatma S. El Sakhawy, Rania A. El Gedaily

**Affiliations:** a Department of Pharmacognosy, Faculty of Pharmacy, Cairo University Kasr Al-Aini St. Cairo 11562 Egypt ahmed.abobakr@pharma.cu.edu.eg; b Clinical and Chemical Pathology Department, Faculty of Medicine Cairo University Al-Saray St. El Manial Cairo 11956 Egypt

## Abstract

*Pseudobombax ellipticum* is native to South America and is cultivated worldwide mostly for its medicinal benefits. The plant is used traditionally in respiratory disorders such as dry cough, in the treatment of fever and stomach pain, and as an antimicrobial and analgesic. The antisickling and antioxidant effects of the flowers of *P. ellipticum* (Kunth) Dugand (red) and *P. ellipticum* cultivar alba (white) were compared using an *in vitro* assay in 2% sodium metabisulfite sickling induction model, DPPH, and metal chelation assays. Both red and white flowers exhibited antioxidant and antisickling activities. In DPPH assay, lower IC_50_ (34.89 ± 0.98 and 53.28 ± 1.14 μg mL^−1^) in red and white flowers respectively were detected relative to Trolox as a positive control (56.82 ± 0.87 μg mL^−1^). Comparable metal chelation activity (81.4 and 77.8 μM EDTA equivalent/mg) was detected in red and white flowers of both cultivars respectively. The average readings of the “reversal of sickling test “revealed a decrease in sickling percent from 49% to 15% in red flowers and to 18% in white flowers. Also, polymerization inhibition rate was increased from 0.34 to 1 and to 0.92 in red and white flowers respectively. Total phenolics, flavonoids and anthocyanins were quantified in red and white flowers as (163.9, 43.13 mg gallic acid equivalent/g extract), (71.92, 34.5 mg rutin equivalent/g extract) and (127.0, 85.9 mg pelargonidine-3-mono glucoside equivalent/kg extract), respectively. Liquid chromatography mass spectrometry (LC-MS) analysis was further employed for detection and identification of anthocyanins in flower extracts. Eight new anthocyanins were identified for the first time in genus *Pseudobombax*. These results reveal the potential role for both red and white flower extracts as possible antisickling agents in sickle cell anemia management.

## Abstract 2

This study revealed the potential antisickling activity of *Pseudobombax ellipticum* cultivars with their metabolite profiling using LC-MS for the first time.

## Graphical abstract

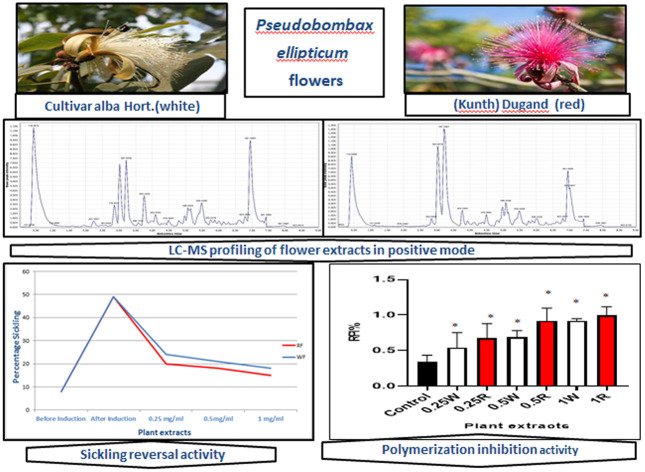

## 1. Introduction

The family Bombacaceae is a small family of flowering plants termed the Kapok family, referring to the cottony white fibers (bombax or kapok) surrounding their seeds.^1,2^ They are characterized by their unique brush-like flowers in late winter.^1^ Members of this family are used traditionally for the management of respiratory illnesses, gastrointestinal ailments and diabetes.^3,4^ The genus *Pseudobombax* comprises 30 species; *P. ellipticum* (Kunth) Dugand also known as *Bombax ellipticum* is cultivated in Egypt.^5^ Its flowers show beautiful 5-inch long red (*P. ellipticum* (Kunth) Dugand) or white (*P. ellipticum* cultivar alba Hort.) stamens, looking very much like a shaving brush.^3^ Few reports were found concerning the biological activities and chemical composition of *P. ellipticum*. Aqueous and ethanol extracts of the cortex exhibited significant antioxidant activity.^6^ From its stem bark, β-lupeol was isolated and showed potent gastro-protective effect.^7^ Cyanidin-3,5-diglucoside was isolated from the flowers.^2^ Sickle cell anemia (SCA) is the most common genetic blood disorder worldwide.^8^ According to estimates, Africa accounts for 75–85% of children born with SCA. Hemoglobin S (HbS) is produced instead of normal adult haemoglobin due to a mutation in the haemoglobin beta gene (HBB).^9^ Normal RBCs have a 120 day lifespan and a flexible biconcave disk-like shape that facilitates easy passage through the microvasculature^10^ while under hypoxic conditions, HbS polymerizes, resulting in “sickle cells,” hard and deformed RBCs with shortened life span (10–20 days) that cause decreased microcirculation and hemolysis.^11^ Hence, antioxidants are very important in SCA management.^12,13^ Pharmaceuticals that are now available for treating SCA are either not sufficiently effective, not entirely safe, or too expensive warranting for developing effective, safe, and affordable drugs from natural sources.^14^ Interestingly, *Ceiba pentandra* (L.) Gaertn (*Bombacaceae*) is used traditionally in Congo to treat SCA and it's aqueous bark extract exhibited significant antisickling and antithrombing activities.^15^ Also, previous reports attributed the antisickling effect of several plants to their high anthocyanin content, present mainly in the flowers.^16,17^ As little data was available concerning *P. ellipticum* flowers phytochemical makeup especially anthocyanins, and further prompted by the well reported anthocyanins antisickling potential, this study aims to identify phytoconstituents especially anthocyanins of potential effects in SCA using LC/MS. The antioxidant and antisickling activities of both *P. ellipticum* red flowers (PR) as well as *P. ellipticum* cultivar alba white flowers (PW) were compared in relation to their metabolite fingerprint.

## 2. Material and methods

### 2.1. Blood samples

The blood utilized in this study was drawn from patients known to have sickle cell anemia (Hemoglobin SS) from the hematology clinic at Cairo University Children Hospital “Abu L-Reesh Mounira”. Informed consents were obtained from human participants of this study and all experiments were approved by the national ethic committee (No. MP3122) at Cairo University. Blood samples withdrawn from the selected patients were confirmed to contain homozygous (SS) red blood cells by haemoglobin electrophoresis carried out on cellulose acetate at pH 8.5. Samples were kept at 4 °C once they were determined that they included SS red blood cells.^16^

### 2.2. Plant material

*P. ellipticum* red flowers (PR) and *P. ellipticum* cultivar alba white flowers.

(PW) were collected from Mostafa El Abed botanical garden, during May 2020. Dr Mohamed El Gibali, Senior Botanist & Consultant at Orman Botanic Garden, Giza, Egypt, kindly carried out the plant identification. A vouchered specimen of the plant, with serial number (20.09.2022) was kept at the Pharmacognosy Department, Faculty of Pharmacy, Cairo University.

### 2.3. Preparation of flower extracts for antisickling assay

For preparation of extracts, fresh flowers (50 g) from each plant were dissected by scissors to small pieces and separately extracted by 200 mL 100% methanol in 1 : 4 solid/solvent ratio using ultrasonic for 2 h. Extracts were then filtered *via* cotton pad and evaporated using rotavapor at temperature of 30 °C that yielded 3 g residue for red flowers and 2.8 g residue for white flowers which were kept till further anaylsis.^18^

### 2.4. Preparation of anthocyanins enriched extracts (AEE)

For another (50 g) of fresh flowers, 200 mL acidified methanol (0.1% formic acid in methanol) was used in a ratio of 1 : 4 solid/solvent and extraction was done in the dark, in amber colored bottles kept in the refrigerator to avoid against anthocyanins degradation. Extraction was done for 3 days followed by sonication for 10 minutes prior to filtration. Extract was evaporated using rotavapor at temperature of 30 °C to yield 2.13 g residue for red flowers and 1.75 g residue for white flowers and kept at −20 °C till further analysis.^18,19^

### 2.5. Total phenolics assay

**Fig. 1:**
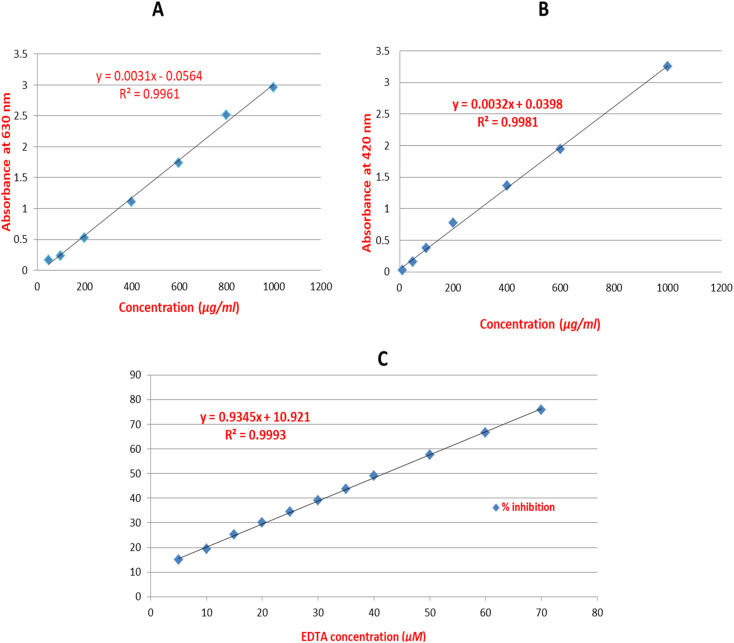
The established calibration curves with the extracted linear regression equations for gallic acid (A), rutin (B) and EDTA (C) standards.

Total phenolics were measured in flower samples using Folin-Cioacalteu assay.^20^ A stock solution of 1 mg mL^−1^ of gallic acid in methanol was prepared as the standard, and seven serial dilutions were made at concentrations of 1000, 800, 600, 400, 200, 100, and 50 μg mL^−1^. Samples from the AEE of white and red flowers were prepared in a concentration of 4 mg mL^−1^ in methanol. Multimode plate reader (Fluostar Omega, BMG LABTECH, Germany) was used to record the outcomes. Six replicates of the 7 standards and 2 samples were pipetted into the wells of the plate measured at 630 nm. The average of the absorbance readings of the 6 replicates for gallic acid standard was recorded and calibration curve was established. From the established calibration curve, the linear regression equation was extracted for gallic acid standard (Fig. 1A). By substitution in the linear regression equation, the total phenolics were detected.

### 2.6. Total flavonoids assay

Total flavonoids were determined using colorimetric determination of flavonoids – AlCl_3_ complex.^21^ A stock solution of 1 mg mL^−1^ of rutin in 100% methanol was prepared as the standard, and seven serial dilutions were made at concentrations of 1000, 600, 400, 200, 100, 50 and 10 μg mL^−1^. Multimode plate reader (Fluostar Omega, BMG LABTECH, Germany) was used to record the outcomes. Six replicates of the 7 standards and 2 samples were pipetted into the wells of the plate measured at 420 nm. The average of the absorbance readings of the 6 replicates for rutin standard was recorded and calibration curve was established. From the established calibration curve, the linear regression equation was extracted for rutin standard (Fig. 1B). By substitution in the linear regression equation, the total flavonoids were detected.

### 2.7. Total anthocyanins assay

Using potassium chloride (0.025 M, pH 1.0) and sodium acetate (0.4 M, pH 4.5) buffer systems, the total anthocyanins content was measured using the pH-differential method.^22^ Each buffer solution (9 mL) was combined with 1 mL of AEE of red and white flowers. The absorbance (Abs) was determined using UV/Vis spectrophotometer, Jenway, England set at 520 and 700 nm, respectively.

### 2.8. Liquid chromatography-mass spectrometry (LC-MS) profiling

An Agilent LC-MS system composed of an Agilent 1290 Infinity II UHPLC coupled to an Agilent 6545 ESI-Q-TOF-MS in positive mode was used to obtain Ultra-high-performance liquid chromatograms. Aliquots (1 μL) of methanolic extracts (5 mg mL^−1^ in MeOH) were analyzed on a Kinetex phenyl-hexyl (1.7 μm, 2.1 × 50 mm) column. Isocratic elution of 90% A (A: 100% H_2_O + 0.1% formic acid) for 1 min followed by 6 min linear gradient elution to 100% B (95% MeCN + 5% H_2_O + 0.1% formic acid) with a flow rate of 0.4 mL min^−1^. ESI conditions were set with the capillary temperature at 320 °C, source voltage at 3.5 kV and a sheath gas flow rate of 11 L min^−1^. Ions detected in the full scan at an intensity above 1000 counts at 6 scans per s, with an isolation width of 1.3 ∼ *m*/*z*, a maximum of 9 selected precursors per cycle and using ramped collision energy (5× *m*/*z*/100 + 10 eV). Purine C_5_H_4_N_4_ [M + H]^+^ ion (*m*/*z* 121.050873) and hexakis (1*H*,1*H*,3*H*-tetrafluoropropoxy)-phosphazene C_18_H_18_F_24_N_3_O_6_P_3_ [M + H]^+^ ion (*m*/*z* 922.009798) were used as internal lock masses.^23^ Metabolites were identified based on their retention times, molecular formulae and their fragmentation patterns, compared to earlier reported data aided with GNPS spectral library search and Sirius.

### 2.9. Antioxidant activity

#### 2.9.1. 2,2-Diphenyl-1-picrylhydrazyl (DPPH) method

One of the most popular techniques for evaluating antioxidant activity is DPPH based in color change in DPPH solution to turn from deep violet color to light yellow color during the reaction. The EC50 of the compounds is used to display the assay's results.^24^ In order to determine a range of concentrations within which the inhibitory concentration 50 (IC_50_) lies, initial solutions of red and white flowers AEE were prepared at concentration spanning from 1000 and 100 μg mL^−1^ in methanol. Extracts that exceeded 50% inhibition in any of the initial screening step, concentrations were serially diluted to provide 5 concentrations. Briefly, 100 μL of freshly prepared DPPH reagent (0.1% in methanol) were added to 100 μL of the samples (dissolved in methanol) in 96 wells plate (*n* = 6), the reaction was incubated for 30 min in dark at room temperature. Decrease in DPPH colour intensity was assessed at 540 nm using a Multimode plate reader (Fluostar Omega, BMG LABTECH, Germany). Trolox was used as standard prepared at 100, 80, 60, 50, 40, 20 and 10 μg mL^−1^. The results of six separate trials were combined to produce a mean and standard deviation (SD) for each sample. By converting the concentrations to their logarithmic value and choosing the nonlinear inhibitor regression equation log(inhibitor) *vs.* normalised response (variable slope equation), the data were analyzed using Microsoft Excel®, and the IC_50_ value was determined using GraphPad Prism 5®.^25^ The IC_50_ was determined from preliminary screening to be less than 100 μg mL^−1^. For each sample, the following serial dilutions were created: 100, 80, 60, 50, 40, 20 and 10 μg mL^−1^.

#### 2.9.2. Metal chelation assay

Metal chelation assay is based on that ferrozine (3-(2-pyridyl)-5,6-diphenyl-1,2,4-triazine-*p*,*p*0-disulfonic acid monosodium salt hydrate) can bind to Fe^2+^ ions to produce a blue color. The iron-ferrozine complex's blue color is attenuated upon binding of compounds and/or extracts to iron (Fe^2+^). Using EDTA as a positive control, the colour reduction is used to gauge the compound's iron chelating action.^26^ Serial dilutions (11) of 0.1 mM EDTA stock solution in water were created at concentrations of 5, 10, 15, 20, 25, 30, 35, 40, 50, 60, and 70 μM. Samples of enriched extracts from red and white flowers were prepared at a concentration of 0.625 mg mL^−1^ in methanol. The assay was conducted using the Santos *et al.* method,^26^ with a few minor adjustments for microplates. In a 96-well plate (*n* = 6), 20 μL of freshly made ferrous sulphate (0.3 mM) was combined with 50 μL of the samples. Then, each well received 30 μL of ferrozine (0.8 mM). For 10 minutes, the reaction mixture was incubated at room temperature. The reduction in the intensity of the generated colour was measured at 562 nm and percentage inhibition was calculated using this formula = (average absorbance of blank − average absorbance of the test/average absorbance of blank) × 100. The multimode plate reader (Fluostar Omega, BMG LABTECH, Germany) was used to record the results. From the established calibration curve plotted from % inhibition of different concentrations of EDTA standard, the linear regression equation was extracted (Fig. 1C) and the results of the samples were presented as μM EDTA equivalent/mg sample. After subtraction from the blank (reagent + solvent), the average of the readings of the 6 replicates was taken.

### 2.10. Biological assay

#### 2.10.1. Sickling reversal test

Using previously established methods, the plants' capacity to reverse the sickling status of the RBCs was determined.^16,27^ Sickle cell blood was washed twice using five times the volume of phosphate-buffered saline (PBS) with a pH of 7.4 by centrifugation at 4000 rpm for three minutes and the flower's total extracts were resuspended in absolute ethanol in a concentration of (0.25, 0.5 and 1 mg mL^−1^). For the control test, 50 μL of washed blood (WB) were added to 950 μL PBS into a clean Eppendorf tube and to 450 μL PBS and mixed with 500 μL of freshly prepared 2% sodium metabisulfite into another clean one. For the inhibition test, 50 μL of WB were added to 400 μL PBS followed by 50 μL from the flower's total extracts and mixed with 500 μL of freshly prepared 2% sodium metabisulfite into clean Eppendorf tubes. Control Tubes represent the initial percentage of sickling and sickling percentage after induction with sodium metabisulfite respectively. Ten μL from each mixture were spotted on a microscope slide covered with a cover slip. To seal the edges of the cover slip, paraffin wax was carefully applied and thus completely preventing air entry. By counting normal and sickled red cells under a 40× light microscope, the ability of the flowers extracts to reverse the sickling of RBCs was observed and documented after incubation of all slides for 2 h at 37 °C. Morphologically, the red blood cells were classified as normal or sickled by observing their shapes. The elongated, C-like, or wrinkled shapes were considered sickled while biconcave or rounded shapes were taken to be normal. Using the following formula, the percentage sickled red blood cells was calculated: Red blood cells percent of sickling (%) = (number of counted sickled red blood cells divided by the total number of counted red blood cells) × 100. For each concentration, the experiment was conducted three times.

#### 2.10.2. Polymerization inhibition test

Following a previous procedure, the polymerization inhibition test was conducted.^11^ In this procedure, the turbidity of the polymerizing solution of RBCs at a wavelength of 700 nm at a temperature of 26 °C was measured. After adding 0.88 mL of freshly made 2% sodium metabisulfite, into a clean cuvette, 0.1 mL of PBS and 0.02 mL of sickled blood, which had been washed twice in five volumes of PBS (1 mL of blood in 5 mL of PBS) and then resuspended in PBS at a ratio of 1 : 1 was added. This serves as control test. For the inhibition test, 0.1 mL of PBS was substituted with 0.1 mL of the flower's total extracts that also were resuspended in absolute ethanol in a concentration of (0.25, 0.5 and 1 mg mL^−1^). For 60 minutes, the absorbance was measured at 700 nm immediately and at periodic intervals. Using the following formula, the rate of polymerization in percentage was computed: rate of polymerization (*R*_p_) = [(final absorbance − initial absorbance)/60] × 100. For each concentration, the experiment was repeated three times. At time zero (*i.e.*, immediately after addition of sodium metabisulfite), the initial absorbance of the polymerizing solution of sodium metabisulfite was measured and subtracted from the final absorbance taken at the end of 60 min. Dividing the resulting value by 60 gives the rate of polymerization inhibition. The statistical significance of difference was calculated by GraphPad Prism 9.5.1 using analysis of variance where value of *p* < 0.05 was considered significantly different.

## 3. Results

### 3.1. Total phenolics, flavonoids and anthocyanins determination

Total phenolics expressed as gallic acid/equivalent were detected at 163.9 ± 6.05 and 43.13 ± 4.2 mg g^−1^ in red and white flower extracts, respectively. Likewise, higher flavonoids level expressed as rutin/equivalent was detected in red flowers at 71.92 ± 4.0 *versus* 34.5 ± 3.8 mg g^−1^ extract in white flowers. Considering the abundance of anthocyanins in flowers, their levels were estimated as mg pelargonidine-3-mono glucoside. The total anthocyanins content was detected at 127.0 and 85.9 mg kg^−1^ in red and white flowers, respectively.

### 3.2. Liquid chromatography-mass spectrometry (LC-MS) profiling of flower extracts

**Fig. 2:**
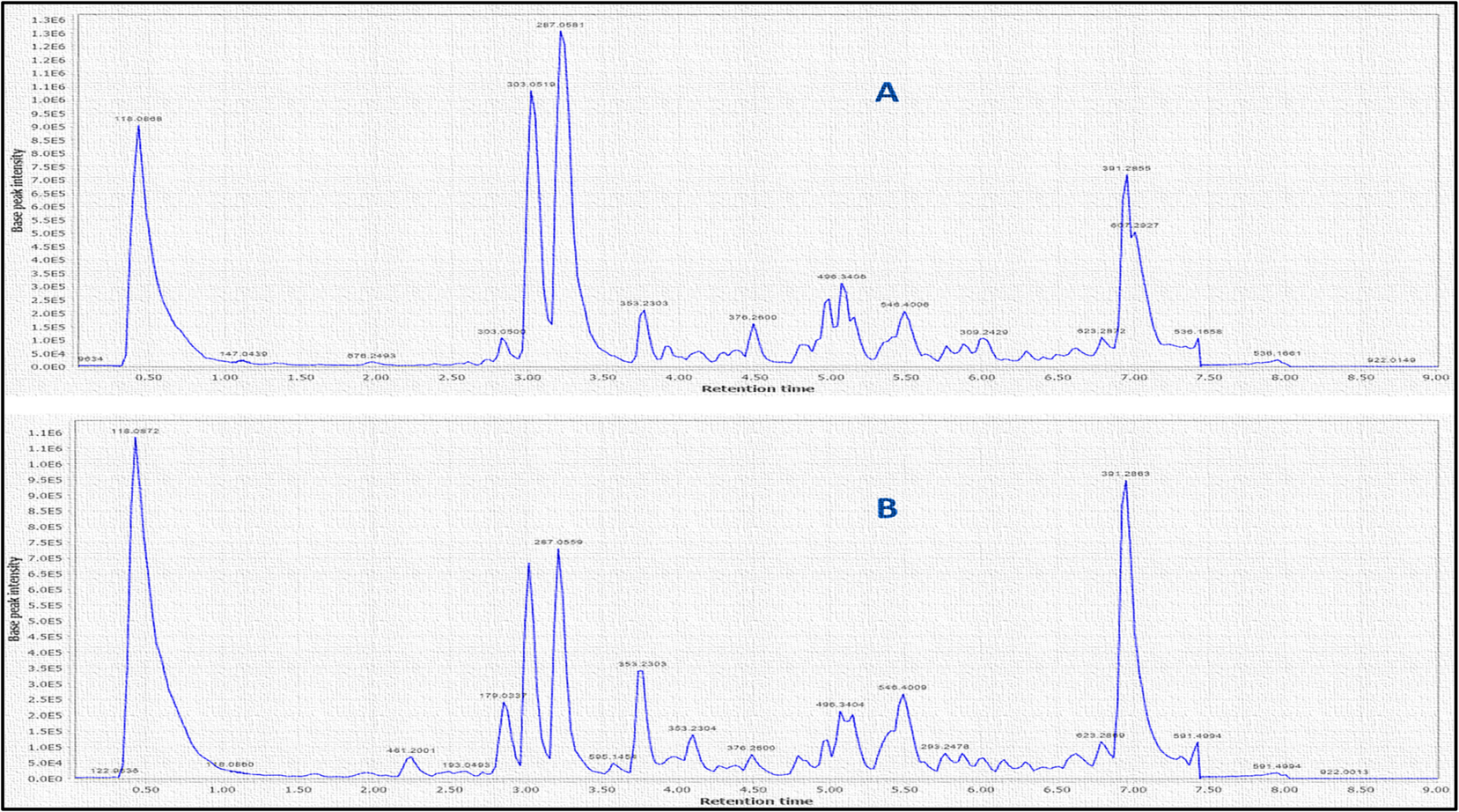
Base peak chromatograms of LC/MS analysis of *P. ellipticum* (Kunth) Dugand (A) and *P. ellipticum* cultivar alba Hort. (B) flower's anthocyanins enriched extracts (AEE) in positive mode on an Agilent LC-ESI-Q-TOF-MS instrument.

**Fig. 3:**
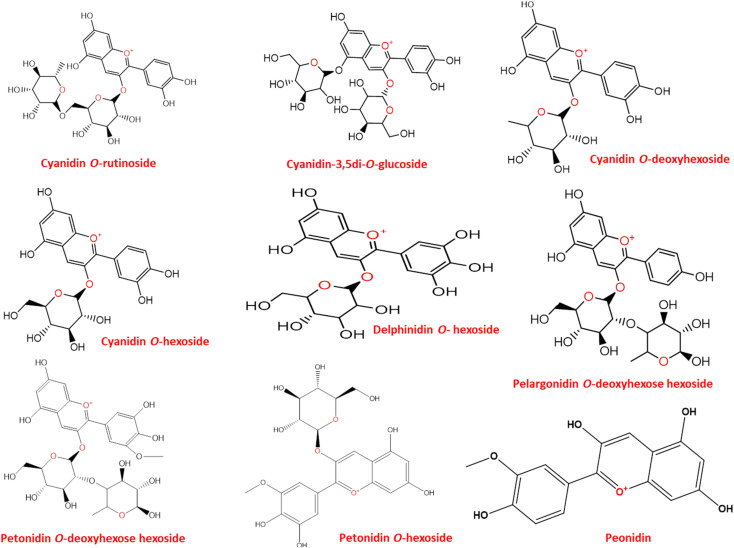
Structures of identified anthocyanins in *P. ellipticum* (Kunth) Dugand and *P. ellipticum* cultivar alba Hort. flower's anthocyanins enriched extract (AEE) in positive mode on an Agilent LC-ESI-Q-TOF-MS instrument.

LC-MS was further used to identify metabolites, mainly anthocyanins as depicted in (Fig. 2). A total of 10 peaks were identified in both flowers (Table 1). Of the 9 anthocyanins detected (Fig. 3), only cyanidin-3,5-di-*O*-glucoside was previously identified.^2^ Cyanidin-3,5-di-*O*-glucoside (peak 2, 611 MS1 *m*/*z*), cyaniding *O*-hexoside (peak 5, 449 MS1 *m*/*z*), cyaniding *O*-rutinoside (peak 6, 595 MS1 *m*/*z*) and cyanidin *O*-deoxyhexoside (peak 7, 433 MS1 *m*/*z*) produced a characteristic fragment ion at *m*/*z* 287 after the loss of a diglucose moiety, a hexose moiety, (a deoxyhexose and hexose) moiety and a deoxyhexose moiety, respectively.^28,29^ The 100% abundance of *m*/*z* at 287 indicated it as cyanidin aglycone in these glycosidic conjugates. Another aglycone was detected in (peak 1, 579 MS1 *m*/*z*) with a fragment ion at *m*/*z* 271 after the loss of a (deoxyhexose and hexose) moiety. The *m*/*z* at 271 with 100% intensity indicates pelargonidin aglycone leading to peak 1 identification as pelargonidin *O*-deoxyhexose hexoside.^28^ Delphinidin *O*-hexoside (peak 3, 465 MS1 *m*/*z*) yielded a fragment ion at *m*/*z* 303 resulting from the loss of hexose moiety.^30^ The *m*/*z* at 303 with 100% intensity indicates delphinidin aglycone. Petonidin *O*-hexoside (peak 8, 479 MS1 *m*/*z*) and petonidin *O*-deoxyhexose hexoside (peak 9, 625 MS1 *m*/*z*) produced a fragment ion at *m*/*z* 317 after the loss of a hexose moiety and (a deoxyhexose and hexose) moiety, respectively.^28^ The *m*/*z* at 317 with 100% intensity indicates petunidin aglycone. Finally, peonidin (5-methoxycyanidin) (peak 10, 301 MS1 *m*/*z*) yielded a fragment ion at *m*/*z* 286 resulting from the loss of a methyl group.^30^

**Table 1:** Identified anthocyanins in *P. ellipticum* (Kunth) Dugand and *P. ellipticum* cultivar alba Hort. flower's anthocyanins enriched extract (AEE) in positive mode on an Agilent LC-ESI-Q-TOF-MS instrument

Peak no.	*R*_*t*_ time (min)	[M + H]^+^	Fragments *m*/*z*	Molecular formula	Identification	Error PPM
(1)	2.07	579.1501	271.0583, 163.0378, 139.0387, 127.0385	C_27_H_31_O_14_	Pelargonidin *O*-deoxyhexose hexoside	−7.1
(2)	2.74	611.1609	287.0551, 85.0283, 71.0492	C_27_H_31_O_16_	Cyanidin-3,5-di-*O*-glucoside	−0.5
(3)	2.83	465.1033	303.0500, 85.0282	C_21_H_21_O_12_	Delphinidin *O*-hexoside	−2.9
(4)	2.84	487.28 [M + Na]^+^	325.0311, 324.0235, 185.0418	C_21_H_20_O_12_	Delphinidin *O*-hexoside	−1.7
(5)	2.88	449.1083	287.0551, 85.0278	C_21_H_21_O_11_	Cyanidin *O*-hexoside	−6.5
(6)	3.13	595.1664	287.0549, 85.0283, 71.0490	C_27_H_31_O_15_	Cyanidin *O*-rutinoside	−3.9
(7)	3.21	433.1134	287.0551	C_21_H_21_O_10_	Cyanidin *O*-deoxyhexoside	−0.7
(8)	3.27	479.0827	317.0657, 85.0286	C_21_H_18_O_13_	Petonidin *O*-hexoside	−2.5
(9)	3.30	625.1770	317.0654, 85.0281, 71.0490	C_28_H_33_O_16_	Petonidin *O*-deoxyhexose hexoside	−3.6
(10)	3.91	301.0709	286.0473, 258.0520	C_16_H_13_O_6_	Peonidin	−2.1

### 3.3. Antioxidant activities

Both red and white flowers showed a potential DPPH radical scavenging activities with IC_50_ value of (34.89 ± 0.98 and 53.28 ± 1.14 μg mL^−1^) respectively relative to Trolox with IC_50_ (56.82 ± 0.87 μg mL^−1^). Red flowers exhibited higher activity than white ones in case of DPPH assay. Likewise, red flowers showed stronger metal chelation effect as another antioxidant action as manifested by 81.45 μM EDTA equivalent/mg sample *versus* 77.8 in case of white flowers.

### 3.4. SCA *in vitro* assays

#### 3.4.1. Sickling reversal test

**Fig. 4:**
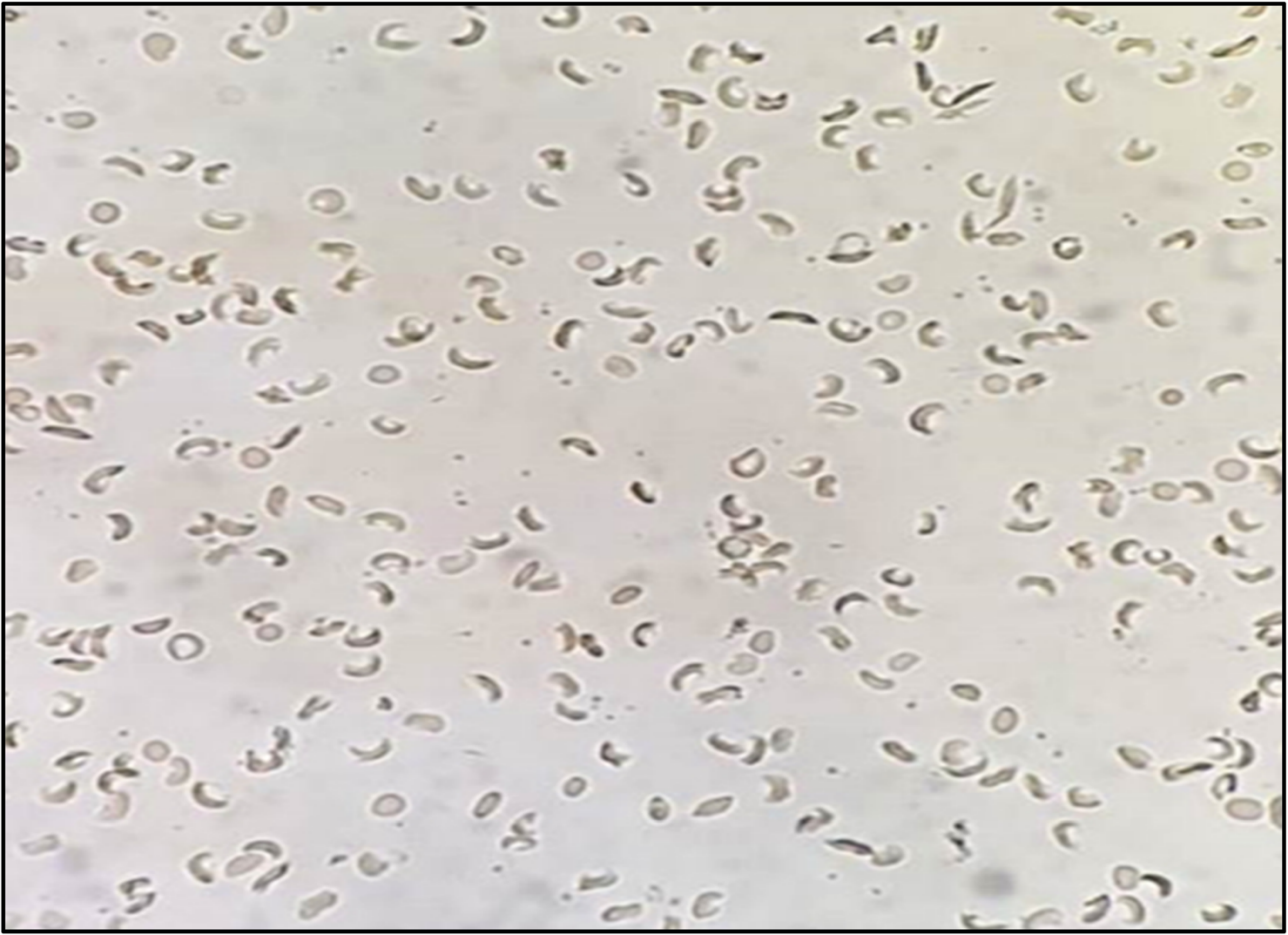
Morphology of sickled RBCs in absence of flowers extracts with approximately 60% elongated C shaped sickled cells.

**Fig. 5:**
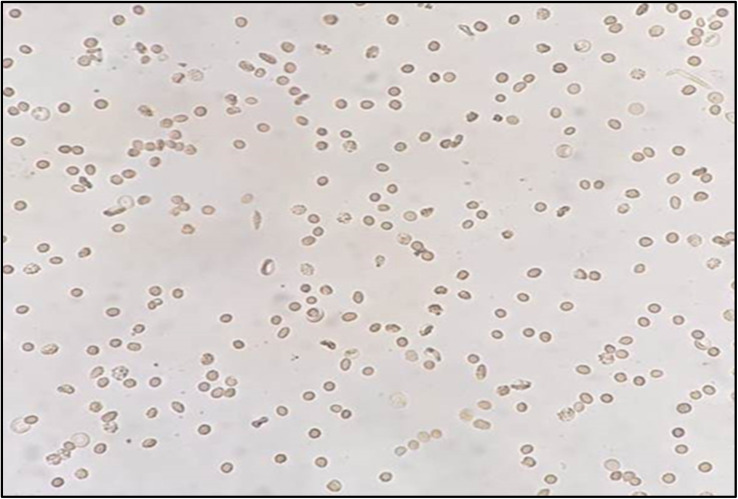
Morphology of recovered RBCs in the presence of PR extract (1 mg mL^−1^) with approximately 90% normal rounded cells.

**Fig. 6:**
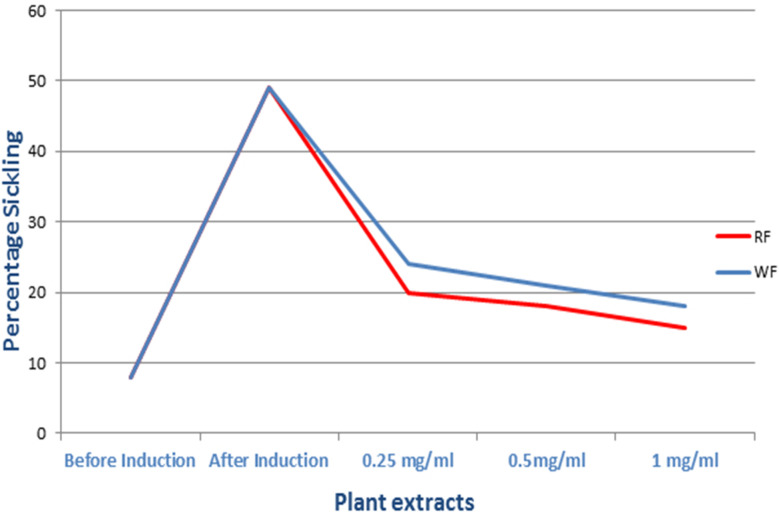
Sickling reversal activity of (PR) and (PW) at different extract concentrations.

Microscopical examination of sickle cells showed elongated or C-like shapes, whilst normal RBCs appeared rounded or biconcave (Fig. 4 and 5). By using sodium metabisulfite, sickled cell percentage increased from 8 to 49%. In contrast, red flowers extracts (0.25, 0.5 and 1 mg mL^−1^) showed decrease in proportion of sickling to 20, 18 and 15% respectively, where white flowers extracts showed 24, 21 and 18% inhibition at a dose of 0.25, 0.5 and 1 mg mL^−1^, respectively (Fig. 6).

#### 3.4.2. Polymerization inhibition test

**Fig. 7:**
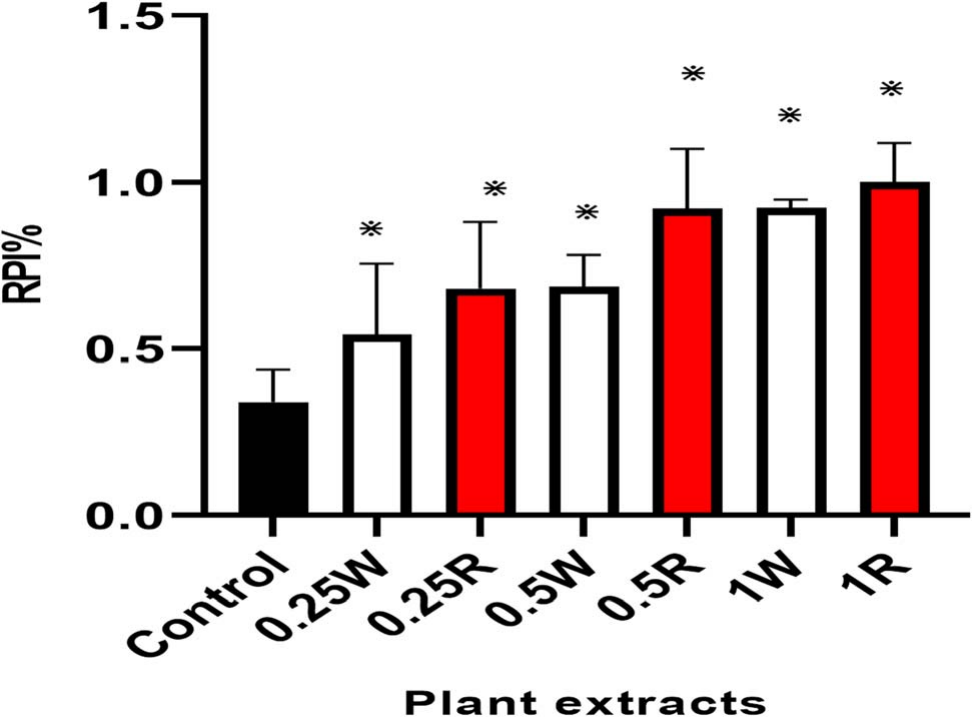
Rate of polymerization inhibition (RPI%) of (PR) and (PW) at different extract concentrations with significant difference relative to control.

PR and PW showed significant increasing in the rate of polymerization inhibition (*P* value = 0.0008). Red flower extracts (0.25, 0.5 and 1 mg mL^−1^) increased the rate of polymerization inhibition to (0.68, 0.92 and 1) respectively, whereas white flower showed 0.54, 0.68 and 0.92 increase in rate at same dose of 0.25, 0.5 and 1 mg mL^−1^ (Fig. 7). The rate of polymerization inhibition of the control was 0.34. Both assays revealed higher efficacy for red flower extract compared to white flower and are in agreement with antioxidant effect.

## 4. Discussion

SCA is a common blood disorder, affecting millions of people worldwide.^8^ Ethno medicinal plants have a great importance in the treatment of various ailments including SCA where, phenolic compounds like flavonoids and anthocyanins appeared to exert potential antisickling activity.^27,31,32^ Anthocyanins especially exhibited promising antioxidant and antisickling activities.^16,17^ Anthocyanins ability to attach to proteins may account for their antisickling effect and to further inhibition of the polymerization of deoxy HbS inside the RBCs.^16^ Anthocyanins also exhibit a stabilizing effect on RBCs membrane and enhance the solubility of deoxy HbS, thereby inhibiting gelification.^16^ In this study, the flowers anthocyanins content was quantified and LC-MS revealed that nine anthocyanins were found enriched in both flowers. Antioxidants are potential management strategies in SCA.^13,27^ Interestingly, the flowers extracts exhibited potential antioxidant activity by DPPH and metal chelation assays. Also, both flower extracts exhibited a potential antisickling and hemoglobin polymerization inhibition activities by preventing or reversing the sickling of RBCs. In accordance with previous reports higher content of active constituents mainly anthocyanins attributed to the higher antioxidant and antisickling activities in PR. As illustrated, PR and PW were rich in phenolics, flavonoids and anthocyanins with a higher content in case of PR bringing them (especially PR) as a promising trial in treatment of SCA.

## 5. Conclusion

The phytoconstituents of PR and PW especially anthocyanins were investigated using LC-MS. Their ethanolic extracts were able to stop or reverse sickling and haemoglobin polymerization of human sickled RBCs, thereby lowering the percentage of sickled cells and the rate of haemoglobin polymerization. Beside the antisickling activity, the extracts exhibited also potential antioxidant activity. These findings may represent a primary screening step highlighting the antisickling potential of the flowers of both cultivars. Further research is needed to detect the compounds responsible for the activity. Also, clinical studies need to be conducted to measure the activity *in vivo* and at the gene level.

## Author contributions

Ahmed S. Mohamed: methodology, software, investigation, writing – original draft Omnia Y. Abd El Dayem: methodology, investigation Ali M. El Shamy: conceptualization, supervision, writing – reviewing and editing Fatma S. El Sakhawy: conceptualization, supervision, writing – reviewing and editing Rania A. El Gedaily: conceptualization, validation, supervision, writing – reviewing and editing.

## Conflicts of interest

The authors declare that they have no known competing financial interests or personal relationships that could have appeared to influence the work reported in this paper.

## Supplementary Material
